# Chemical Composition and Cytotoxic Activity of the Essential Oil and Oleoresins of In Vitro Micropropagated *Ansellia africana* Lindl: A Vulnerable Medicinal Orchid of Africa

**DOI:** 10.3390/molecules26154556

**Published:** 2021-07-28

**Authors:** Md. Moshfekus Saleh-E-In, Paromik Bhattacharyya, Johannes Van Staden

**Affiliations:** Research Centre for Plant Growth and Development, School of Life Sciences, University of KwaZulu-Natal Pietermaritzburg, Private Bag X01, Scottsville 3209, South Africa; saleheinm@kangwon.ac.kr (M.M.S.-E.-I.); paromik600@gmail.com (P.B.)

**Keywords:** *Ansellia africana*, cytotoxic, essential oil, micropropagation, oleoresins, orchid, MTT, Vero cells

## Abstract

Orchids are rich treasure troves of various important phytomolecules. Among the various medicinal orchids, *Ansellia africana* stands out prominently in the preparing of various herbal medicines due to its high therapeutic importance. The nodal explants of *A. africana* were sampled from asymbiotically germinated seedlings on basal Murashige and Skoog (MS) medium and were micropropagated in MS medium supplemented with 3% sucrose and 10 µM *meta* topolin (*m*T) + 5 µM naphthalene acetic acid (NAA) +15 µM indole butyric acid (IBA) + 30 µM phloroglucinol (PG). In the present study, the essential oil was extracted by hydrodistillation and the oleoresins by the solvent extraction method from the micropropagated *A. africana*. The essential oil and the oleoresins were analysed by Gas Chromatography (GC) and GC/MS (Mass spectrometry). A total of 84 compounds were identified. The most predominant components among them were linoleic acid (18.42%), *l*-ascorbyl 2,6-dipalmitate (11.50%), linolenic acid (10.98%) and *p*-cresol (9.99%) in the essential oil; and eicosane (26.34%), n-butyl acetate (21.13%), heptadecane (16.48%) and 2-pentanone, 4-hydroxy-4-methyl (11.13%) were detected in the acetone extract; heptadecane (9.40%), heneicosane (9.45%), eicosane (6.40%), n-butyl acetate (14.34%) and styrene (22.20%) were identified and quantified in the ethyl acetate extract. The cytotoxic activity of essential oil and oleoresins of micropropagated *A. africana* was evaluated by MTT (3-(4,5-dimethylthiazol-2-yl)-2,5-diphenyltetrazolium Bromide) assay on Vero cells compared to the standard drug doxorubicin chloride. The present research contains primary information about the therapeutic utility of the essential oil and oleoresins of *A. africana* with a promising future research potential of qualitative and quantitative improvement through synchronised use of biotechnological techniques.

## 1. Introduction

Medicinal herbs are rich treasure troves of many bioactive compounds which provide important remedies for chronic disorders and ailments [1,2]. With a special focus on Africa, the Traditional African Pharmacopeia provides cheap and economic bio-remedies to large African populations who are economically struggling to survive [3,4]. Among the herbal remedies used in various pharmacopoeias globally, orchids occupy a prominent position due to their various medicinal values in alternative treatment options [5]. Despite the fact that orchids have a huge reservoir of bioactive molecules, many of them are yet to be explored for various pharmaceutical applications; for example, *Dendrobium chrysotoxum*, *Bulbophyllum kwangtungense*, *Dendrobium macraei* and *Cypripedium parviflora.* The pharmacological potentials of orchids were evaluated in other countries, but very few studies have been reported on orchid species in South Africa. 

*Ansellia africana*, popularly known as “Leopard Orchid” is one such species which is highly prized globally because of its multifaceted medicinal attributes [6]. Traditionally, it has been used by various tribes across the African continent, particularly by the Mpika tribe of Zimbabwe and Zulus of South Africa [6,7,8]. Unfortunately, high rates of deforestation and over-collection have severely depleted the natural populations of *A. africana,* making it rare, and the International Union for Conservation of Nature (IUCN) has declared it “vulnerable”. It has been listed in the red list of rare, endangered and threatened (RET) plants [9]. To envisage and unlock the large medicinal uses of this unexplored orchid taxa, planned usage of plant tissue culture-based biotechnological approaches provides a sustainable alternative, sufficing the aspects of conservation as well as commercial exploitation [10,11].

Moreover, variation in the in vitro culture conditions, particularly plant growth regulators (PGRs), remarkably enhances metabolite synthesis. The biologically important compounds of pharmaceutical importance get deposited as a result of in vitro stress [12,13,14]. Traditionally, decoctions and infusions obtained from the roots and stem parts of *A. africana* are used to treat various chronic ailments and have been reported to possess strong antimicrobial activity against various bacterial and fungal pathogen strains [4,15]. Furthermore, *A. africana* extracts were shown to inhibit acetylcholinesterase activity, which may be a promising remedy to Alzheimer’s disease [15]. Although there exists no baseline phytochemical data available on *A. africana,* terpenoids, flavonoids and alkaloidal compounds are responsible for these activities that might exist in the species through proper investigation. Nonetheless, very limited reports exist on pharmacological activities of the different parts of *A. africana.* Keeping in perspective the view about the immense potential of *A. africana*, the present research attempts to decipher the chemical composition and cytotoxic activity of essential oil and oleoresins obtained from micropropagated plants of *A. africana*. Extensive literature surveys revealed that the chemical composition along with cytotoxic activity of the essential oil and oleoresins from the micropropagated plants of *A. africana* have not been reported to date. This is the first approach of a phytochemical study on this orchid species.

## 2. Results

### 2.1. Plant Tissue Culture of A. africana

**Figure 1:**
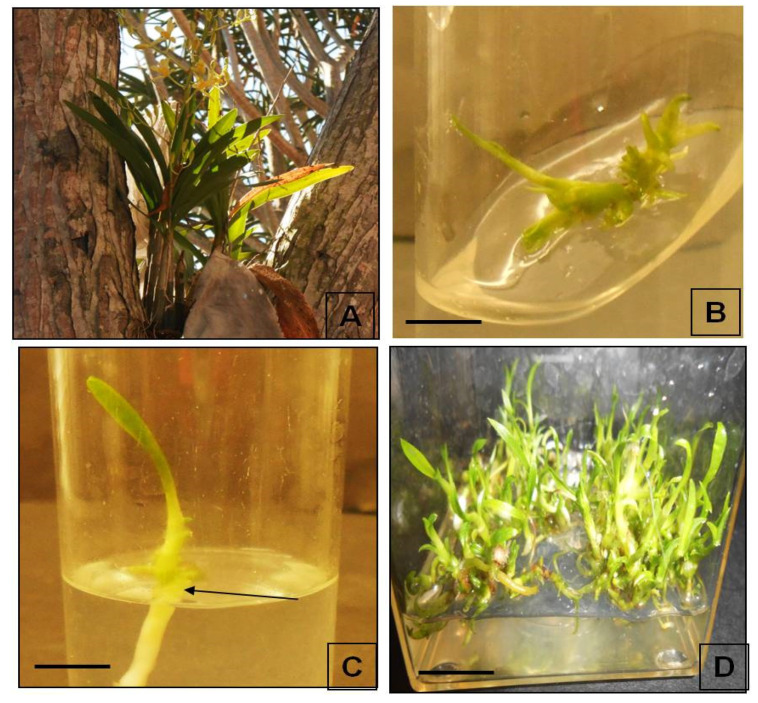
(**A**) Wild plants of *A. africana* in flowering condition, (**B**) culture initiation in MS medium + 10 µM *m*T (bar = 2 mm), (**C**) induction of plantlets and growth in +10 µM *m*T (bar = 2 mm) after 15 days of culture initiation, and (**D**) proliferation of multiple shoots after 30 days when maintained in 10 μM *m*T + 5 μM NAA (bar = 1 cm).

In the sustainable utilization of medicinal plant bio-resources (MAP), plant tissue culture plays a pivotal role. In the fast production of quality planting materials (QPM), plant growth regulators play a major role [16]. In the present research, we have utilized the plant material generated from the aromatic cytokinin-mediated regeneration pathway of *A. africana* as the source material for essential oil extraction [9] (Figure 1). The conducive role of aromatic cytokinin in producing higher secondary metabolites in medicinal plants has been well documented. Our previous research on *A. africana* PLB biomass has provided vital insights into the role of *m*T and its derivatives in the upregulation of phenolic acid contents and subsequent antioxidant activity [12]. Furthermore, the protocol reported by Bhattacharyya et al. [9] ascertained a high degree of clonal fidelity, which is a prerequisite of any plant tissue culture-derived planting material [17]. Within a short time, genetically stable plant material was obtained for *A. africana,* which was further processed to extract essential oil.

### 2.2. Chemical Composition of the Essential Oil and Oleoresins

**Figure 2:**
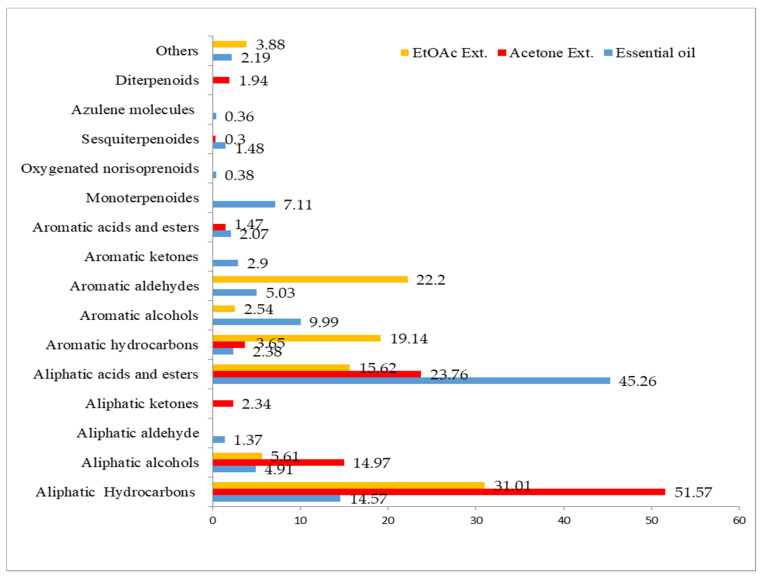
Major identified compounds (in %) of micropropagated plants of *A. africana*.

The highest yield of extract was obtained from acetone extract, with 3770 mg (dry weight basis), followed by essential oil (10 mg) and ethyl acetate extract (5.3 mg) (fresh weight basis) in 100 g material. The colours of acetone, ethyl acetate and essential oil are brown gummy, transparent and slightly yellowish, respectively. The results of GC-MS analyses of essential oil, acetone and ethyl acetate extracts of *A. africana* are summarized in Table 1 and Figure 2.

The presence of aliphatic hydrocarbons, acids and esters are the characteristic feature of the samples under investigation. The highest amounts of aliphatic acids and esters were found in the essential oil (45.26%), followed by acetone (23.76%) and ethyl acetate extracts (15.62%). On the other hand, acetone extract contained the highest portion of aliphatic hydrocarbons (51.57%) compared to the other two samples—ethyl acetate (31.01%) and essential oil (14.57%). In addition, aromatic aldehydes (22.2%) and hydrocarbons (19.14%) were predominantly found in ethyl acetate extract. Furthermore, aromatic alcohols were present in essential oil (9.99%) and ethyl acetate extracts (2.54%), while monoterpenoides (7.11%) are present in essential oil. Sesquiterpenoides were recorded in minute quantities (1.48%) in essential oil.

**Figure 3:**
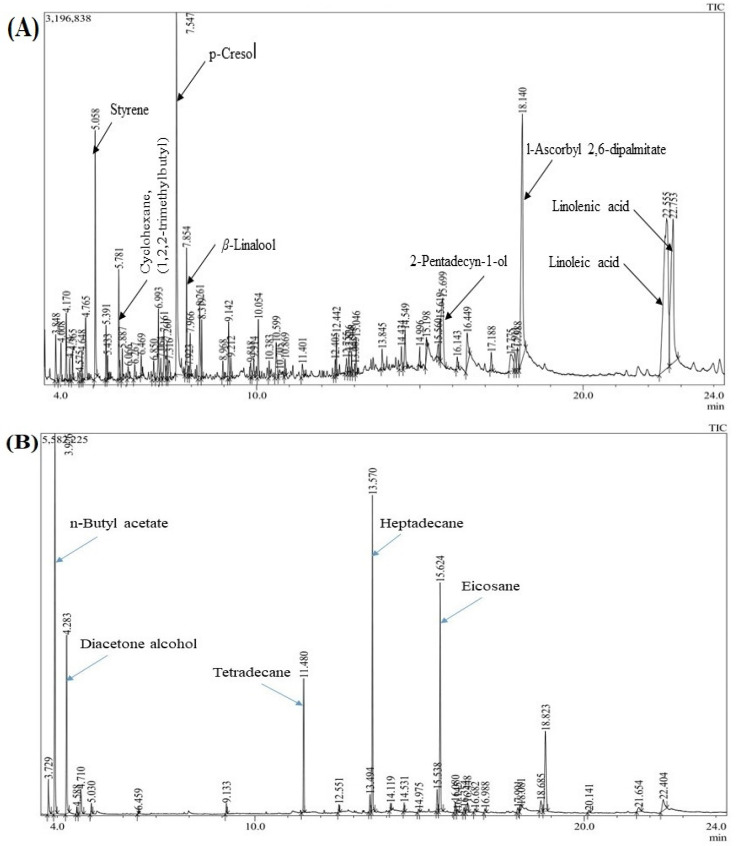

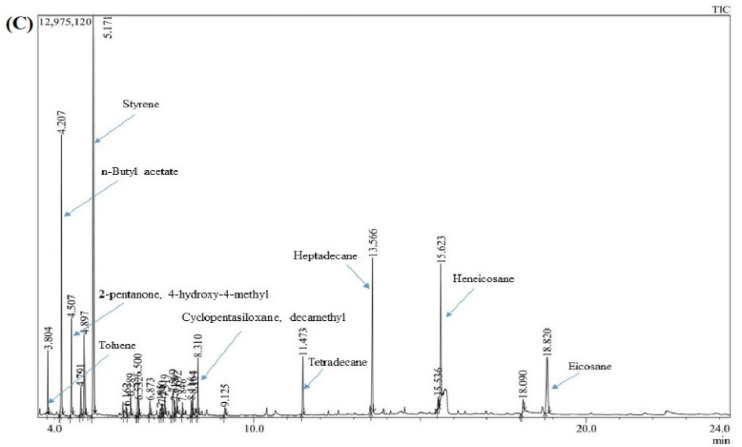
Chromatographic profile of (**A**) essential oil and oleoresins (**B**) acetone and (**C**) ethyl acetate from *A africana* micropropagated plants (details are presented in the Supplementary Figure S1).

**Figure 4:**
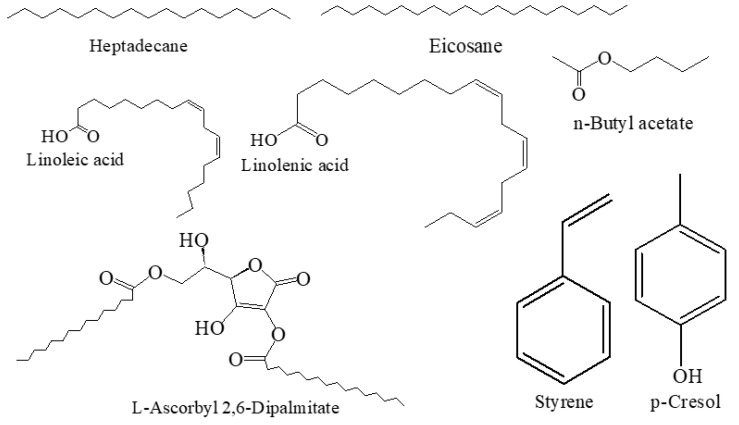
Major identified compounds of micropropagated plants of *A. africana*.

GC-MS analysis showed 55 compounds in the essential oil that constituted 100% of the total oil. Linoleic acid (18.42%) was the major constituent in the oil, followed by *l*-ascorbyl 2,6-dipalmitate (11.50%), linolenic acid (10.98%), erythro-1-phenylpropane-1,2-diol (9.99%), styrene (4.64%), eicosane (3.89%) and 2-pentadecyn-1-ol (3.77%). In the acetone extract, 26 compounds were identified and accounted for 100% of the total extract. It was found to be rich in eicosane (26.34%). The other major components were—n-butyl acetate (21.13%), heptadecane (16.48%) and tetradecane (6.60%). In this study, 26 compounds were also successfully identified from the ethyl acetate extract, which made up 100% of the total amount. The predominant constituents of ethyl acetate extract were styrene (22.20%), n-butyl acetate (14.34%), heneicosane (9.45%), heptadecane (9.40%), eicosane (6.40%), *p*-xylene (5.24%), 2-pentanone, 4-hydroxy-4-methyl (4.79%) and tetradecane (3.83%). Overall, this is the first result on the chemical composition of essential oil and oleoresins from the micropropagated plants of *A. africana*. The GC-MS chromatogram of the predominant components are shown in the plants in Figure 3 (details are presented in the Supplementary Figure S1). The structures of a few major compounds are shown in Figure 4.

### 2.3. PCA Analysis of the Essential Oil and Oleoresins

**Figure 5:**
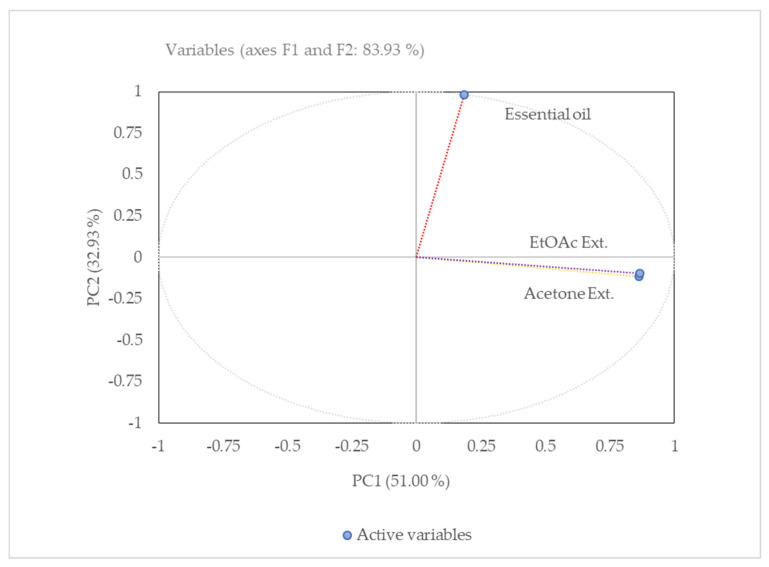
Principal component analysis (PCA) of the correlation between the essential oil and oleoresins composition based on GC–MS results.

The score plot (Figure 5) shows the relationship between different extracts (the observations) and the loading plot shows how strongly each characteristic influences a PC (other variables). The first principal component (PC), PC1, explained nearly 51% of the data variability and the second PC (PC2) explained roughly 32.93%, retaining roughly 83.93% of all variability in the experimental data. Materially, three principal components would explain 100% of the total variation.

### 2.4. The Cytotoxicity of the Essential Oil and Oleoresins

**Figure 6:**
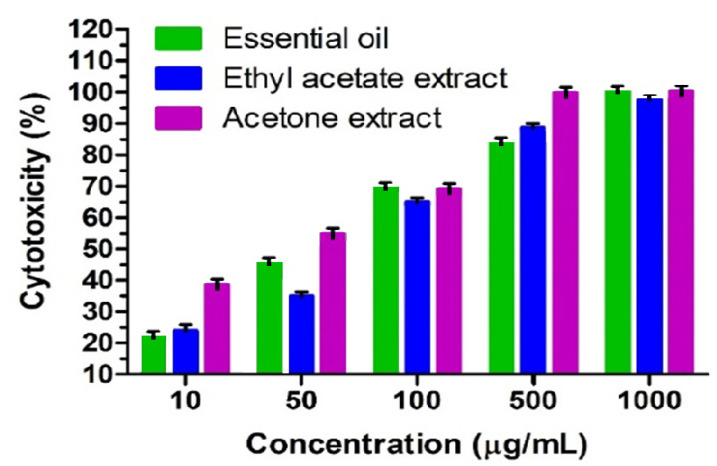
In vitro cytotoxicity percent (%) of *A. africana* essential oil and oleoresins with different concentrations on the Vero cell line by MTT assay. The data represent mean ± standard error of the mean of three independent samples with eight replicates (n = 8), *p* ≤ 0.05.

The cytotoxic activity of the essential oils and oleoresins from the micropropagated *A. africana* on Vero cells is depicted in Table 2. The present investigation is the first report on the cytotoxic effects of essential oil and oleoresins. The essential oil showed cytotoxic activity with an LC_50_ value of 52.53 µg/mL followed by the acetone extract (25.64 µg/mL) and ethyl acetate extract (60.05 µg/mL). However, they were not comparatively as cytotoxic as the anticancer drug doxorubicin hydrochloride (3.46 µg/mL). The essential oil and acetone extract showed the highest cell morality at the dose of 1000 μg/mL (Figure 6). The table shows that the cytotoxic activity of the essential oil differed fractionally with respect to the ethyl acetate extract, however, the acetone extract showed a significant difference in activity with respect to the essential oil and ethyl acetate extract.

## 3. Discussion

Plant tissue culture techniques offer an effective alternative for the regulated production of pharmaceutically important secondary metabolites under a controlled laboratory atmosphere with special reference to the essential oil production [18,19]. Furthermore, in vitro propagation-based approaches are also used to produce mass production of chemically stable, metabolically rich and economically profitable plants [20]. The present research further validates the conducive role of an in vitro propagation module in the quantitative production of the twelve major entities (namely *p*-cresol, styrene, linoleic acid, heptadecane, tetradecane, eicosane, heneicosane, 2-pentanone, 4-hydroxy-4-methyl, *l*-ascorbyl 2,6-dipalmitate and n-butyl acetate) along with other non-major components, which are in close synergy with micropropagated *Lavandula dentate* essential oil. The report showed that IBA could be able to increase the essential oil composition in micropropagated *L. dentate* [21]. Similarly, in in vitro propagated *Ajuga bracteosa* shoots, remarkable changes in the essential oil yield were reported mainly in the levels of α-terpinene, γ-terpinene and terpinolene when treated with varying concentrations of IBA [22] Like-wise, *m*T treatment has also been reported to influence the chemical composition of important MAPs like phytol [23]. In addition, Ali et al. [22] reported the positive correlation between the biosynthesis of volatile organic compounds and in vitro shoot regeneration in the different developmental stages of *A. bracteosa*. Nonetheless, to date, the exact mechanism is not fully understood with respect to the biosynthesis of volatile compounds that are enhanced under PGR treatments. Hence, further investigations are needed to understand the biosynthesis mechanism of the essential oil compounds under PGR treatment.

The GC-MS results revealed that the essential oil and oleoresins constituted a complex mixture of aliphatic and aromatic hydrocarbons, alcohols, aldehydes, ketones, azulenes, acids and esters, terpenoids such as monoterpenoides, oxygenated norisoprenoids, sesquiterpenoides, diterpenoids along with other miscellaneous constituents. The identified major constituents were also found in other plant species that exhibited close synergy with present research findings. In this context, 2-pentanone, 4-hydroxy-4-methyl (diacetone alcohol) has been identified in *Stachys laxa* (12.3%) essential oil [24] which was found to be similar (11.13%) in acetone extracts. In addition, eicosane and heneicosane were identified as high amounts (24.0 and 10.9%) in the aerial parts of *Micromeri ajuliana* [25], and eicosane was a major constituent in the essential oil of the leaves of *Barringtonia asiatica* (27.4%) [26] which was found to be as high at 26.34% and 9.45% in the current study, respectively. Besides, heneicosane was also identified at 8.4–14.1% in in vivo and in vitro cultures of *Caryopteris* species [27] On the other hand, the GC-MS analysis of the leaf oils of *Elephantopus scaber* L. (from 12 locations in southern China) led to the identification of *n*-tetradecane (1.19–5.26%) and *n*-heptadecane (2.48–15.32%) as the major compounds with other constituents [28] which can be compared to our present study.

In such an investigation, styrene has been identified in many foodstuffs such as cereals, fruits, coffee beans, olive oil, beer, beef meat and cinnamon. Styrene can be produced from cinnamaldehyde in cinnamon by an oxidative degradation followed by an intermediate stage of cinnamic acid via decarboxylation. The WHO-recommended level of styrene is 7.7 μg/kg body weight per day for an adult as a tolerable daily intake [29] The amount of styrene in cinnamon is high—levels of 40 mg/kg have been reported [30] Furthermore, styrene was also identified in the essential oil of *Herba taxilli* (11.42%) and styrax gums (70.4% and 30.9%) of two different varieties [31,32] and also in *Peganum harmala* smoke and volatile oil (4.2%) [33] which were detected in a significant amount (4.64% and 22.20%) in the current study. In the case of *p*-cresol, it was detected in *Mutellina purpurea* flower essential oil (17.4%) [34] and the volatile extract from rhizomes of *Cnidium officinale* (10.17%) [35] In that case, *p*-cresol is a compound of *A. africana,* which was detected at 9.99% like other volatile constituents. Nonetheless, all the compounds were identified from the different extracts of *A. africana* for the first time by the micropropagation method.

In the context of solvent extraction, the impact of selected aqueous and organic solvents played an important role in the extraction of constituents and their subsequent cytotoxic effect. The extraction efficiency of the phytochemicals highly depends on the polarity index, extraction technique, extraction time and applied temperature. In the current study, we chose three types of solvent and temperature frames to extract the compounds that produced various compositions in the different extracts. The results showed that the aqueous extract had high levels of certain specific compounds which are not found in the acetone or ethyl acetate extract or found in low amounts, and vice-versa. Therefore, the variation of cytotoxic effect was observed in the different extracts. From the GC analysis (Figure 3), some high amount peaks in the extracts can be further isolated for various biological properties such as anticancer properties.

The principal component analysis (PCA) has been performed to reduce the dimensions of the data set, for co-relation, and to discriminate the essential oil and oleoreisns’ chemical compositions. Based on the PCA analysis, the three samples were grouped into two different clusters. Ethyl acetate and acetone extracts were positively co-related in the first principal component PC1, whereas essential oil was negatively correlated in the second cluster of PC2. Graphically, ethyl acetate and acetone extracts that are close to each other in the score plots have similar chemotypes. It was observed that the number of principal components depends on the number of variables, as the percent of total variance increases with a lower number of variables. Usually, the proportion of total variance decreases by increasing the number of variables. Furthermore, ethyl acetate and acetone extracts were clustered together because of the presence of dodecane, tetradecane, heptadecane, eicosane, 2-pentanone, 4-hydroxy-4-methyl, nonadecanol and n-butyl acetate compounds.

According to the plant screening program (in vitro cytotoxic activity) of the American National Cancer Institute (NCI), the limit of toxicity of plant crude extracts at 50% inhibition (IC_50_) of proliferation is less than 20 μg/mL (incubation between 48 and 72 h), while it is set at less than 4 μg/mL for pure compounds [36] Based on NCI, essential oil and oleoresins showed a non-toxic effect in the present investigation, where the percent of cytotoxicity has shown a dose-dependent graph (Figure 6) of the studied samples according to the concentrations. On the other hand, the bioactivity of the essential oil and oleoresins may be responsible for their predominant compounds (Figure 3) or synergistic effect of major and minor compounds due to their reported broad pharmacological activities. Micropropagated plants of *A. africana* contained a higher percentage of heptadecane, eicosane, n-butyl acetate, styrene, *p*-cresol and fatty acid constituents such as linoleic, linolenic acids and *l*-ascorbyl 2,6-dipalmitate. Although these compounds are the major volatiles in *A. africana*, they were also isolated in pure form in other plant species. These predominant components are reported to have numerous pharmacological uses, especially with cytotoxic and anticancer effects. Heptadecane, eicosane and n-butyl acetate were present as the most predominant component in the oleoresins (acetone and ethyl acetate extracts), and have been reported to have anti-inflammatory and antimicrobial effects [37] cytotoxic effects on HeLa and MCF-7 cell lines, and antimicrobial, larvicidal [38] and cytotoxic [39] properties, respectively. Styrene was also reported as genotoxic, cytotoxic and mutagenic [40,41,42,43], and has been detected in high amounts in ethyl acetate extract in the current investigation. *p*-Cresol and fatty acid constituents of linoleic, linolenic acid and *l*-ascorbyl 2,6-dipalmitate were present in a relatively significant amount in essential oil. *p*-Cresol was widely reported as a cytotoxic compound against different cell lines such as EA.hy926 (EAHY) endothelial and U937 cells, renal epithelial tubular cells, RAW264.7 cells and Bluegill Sunfish BF-2 cells [44,45,46,47].These fatty acids are well-reported for their anticancer and cytotoxic activities, and for their diverse pharmacological actions. Linoleic acid suppressed colorectal cancer cell growth [48] and inhibited the growth of human ovarian carcinoma cells (A2780) and induced mitochondrial-related apoptosis [49] and showed cytotoxicity to Bovine Lens Epithelial Cells [50]. Linolenic acid was reported to have activity against breast cancer [50] and promoted mitochondrial apoptosis for mammary gland chemoprevention [51,52], and was cytotoxic to fresh human tumour cells [53]. Recently, *l*-ascorbyl 2,6-dipalmitate was reported for its antitumour, antibacterial [54] anticarcinogen [55] cytotoxicity [56] and antiproliferative [57] activities. The rich content of linoleic acid, *l*-ascorbyl 2,6-dipalmitate, linolenic acid and erythro-1-phenylpropane-1,2-diol in the essential oil could suggest its potential medicinal value due to their cytotoxic and anticancer properties. The cytotoxic effect of the current study may be due to the major compounds of the essential oil and oleoresins or may be due to synergistic effects of the various compounds in the studied samples at different ratios. The possible mechanism of the cytotoxic activity depends on chemical constituents and experimental cells that are not known at this stage. The mechanisms of the cytotoxic activity can be described in many ways, which depend on the nature of compounds and experimental cells. The essential oils are responsible for some lipophilic compounds that can pass through the cytoplasmic membrane of the cells and degrade its lipoprotein structure [58] Another reason may be cellular toxicity mediated by disturbances in cellular homeostasis of calcium ions, increasing ROS production or unspecific disturbances in the inner mitochondrial membrane of the cells [59]. Further studies are required to unravel the mechanism of action by first isolating and characterizing the bioactive constituents from *A. africana*. This investigation provides useful information for pharmaceutical industries for the formulation of new medicinal or herbal products.

## 4. Material and Methods

### 4.1. Chemicals

Bacteriological Agar powder was purchased from Du Pont de Nemours Int., South Africa and Oxoid Ltd., Basingstoke, Hampshire and England. Naphthalene acetic acid (NAA), Indole-3-butyric acid (IBA), Phloroglucinol (PG), myo-inositol, vitamins (thiamine HCl, nicotinic acid, pyridoxine HCl) and glycine, were obtained from Sigma Aldrich, Germany. *meta*-Topolin (*m*T) was prepared as described previously [60,61]. The compounds 3-(4, 5-Dimethylthiazol-2-yl)-2, 5-diphenyltetrazolium Bromide (Sigma Aldrich, Darmstadt, Germany) Minimal Essential Medium (MEM) (Whitehead Scientific, Winelands Close, Stikland, 7530, South Africa), Gentamicin (Virbac, Carros, France), Foetal Calf Serum (Highveld Biological, Johannesburg, South Africa) and Doxorubicin chloride (Pfizer Laboratories, 85 Bute Lane, Sandton, 2146, South Africa) and Phosphate Buffered Saline (PBS) (Whitehead Scientific, Winelands Close, Stikland, 7530, South Africa) were used. All chemicals used in the assays were of analytical grade.

### 4.2. Micropropagation and Material Generation from A. africana

Mature plants of *A. africana* were sampled from wild habitats and were maintained in the greenhouse of the University of KwaZulu-Natal (UKZN), Pietermaritzburg, South Africa (Figure 1A). Capsules were sampled from these maintained plant germplasms and were germinated asymbiotically on Murshige and Skoog (MS) medium [62] following the protocol described by Vasudevan and Van Staden [63]. From the germinated seedlings of *A. africana*, nodal explants (Figure 1B) were excised and were cultured in MS medium augmented with 3% sucrose (Merck, KGaA, Darmstadt, Germany), 0.8% agar (Oxoid, Hampshire, United Kingdom) and were micropropagated using 10 µM *m*T + 5 µM NAA + 15 µM IBA + 30 µM PG in accordance with the protocol described by Bhattacharyya et al. [9]. The pH of the medium was adjusted to 5.8 before autoclaving at 121 °C for 15 min. For each treatment, 5 replicates were taken, and the experiments were repeated in triplicate. All cultures were maintained at 25 ± 2 °C, 80% RH (Relative Humidity), and 12 h photoperiod at 50 µmol/m^−2^/s^−1^ irradiances provided by cool-white fluorescent tubes (OSRAM, Munich, Germany). Proliferated multiple shoots with roots (Figure 1C,D) were harvested after 10 weeks from the cultures and were used as sample material [9] for subsequent essential oil and oleoresin extraction and chemical profiling.

### 4.3. Extraction

Fresh propagated plant material of *A. africana* was subjected to hydro distillation using a Clevenger-type apparatus for 4 h to obtain the essential oil. Oleoresins (ethyl acetate and acetone extract) were prepared by the solvent extraction method. First, oleoresin was prepared from the emulsified water (residual water part after extraction of the essential oil) by extracting with ethyl acetate solution and dried by vacuum rotary evaporator under high pressure at 35–37 °C. The oil and extracted portion were dried through sodium sulphate. Secondly, another portion of orchid materials was air-dried at room temperature in the shade and subsequently pulverized. The powder material (9.63 g) was then extracted with acetone by Soxhlet apparatus in a temperature controlled (60 to 80 °C) heating mantle for 72 h. The extracted samples were stored at 4 °C until further analyses.

### 4.4. Analysis of the Essential Oils

The essential oils and oleoresins were analysed using GC-2010 and GC-MS QP 2010 SE (Shimadzu Corporation Japan) via Electron Impact Ionization (EI) method, which was fitted with a Zebron ZB5MS-plus (Phenomenex, Inc., Torrance, CA, USA) capillary column. The initial column (30 m × 0.25 mm, 0.25 μm film thickness) oven temperature was 40 °C (hold for 1 min). The injection temperature was 200 °C (hold for 10 min, at the rate of 12 °C/min). The carrier gas was helium at a constant pressure of 72.3 kPa (acquisition parameters set to full scan and start at 50 *m*/*z* and end at 700 *m*/*z*). The split ratio was 5.0, and the ionization energy was 70 eV. The compounds were identified (FID detector) by comparing retention indices with the internal reference mass spectral NIST (National Institute of Standards and Technology) 11 library.

### 4.5. Cytotoxicity Assay

The colorimetric assay of MTT (3-(4,5-dimethylthiazol-2-yl)-2,5-diphenyltetra-zolium Bromide) was used to determine the viable cell growth after incubation of Vero cells with the extracts, according to the method [64,65,66] with slight modification. Minimal Essential Medium (MEM) was used to grow Vero cells supplemented with 0.1% gentamicin and 5% fontal calf serum. Cells of a sub-confluent culture were harvested, centrifuged (at 200× *g*) for 5 min and re-suspended in MEM to 5 × 10^4^ cells/mL. The concentration of the extract was 10 mg/mL. From the mother solution, five concentrations (1, 0.5, 0.1, 0.05 and 0.01 mg/mL) were applied with eight replicates (sample number = 3) to the 96-well plate. The reference drug was used as doxorubicin chloride. The absorbance was measured in a microplate reader (Chromate 4300, Awareness Technology, Palm City, FL 34990, USA) according to the methods. The LC_50_ values were calculated from the linear regression Graph by Microsoft Excel10 version software.

### 4.6. Statistical Analysis

Means, SD, SEM calibration curves (with 95% confidence interval, *p* ≤ 0.5) and linear regression analyses (R2) were determined using Microsoft Excel 2016 (Microsoft Corporation, Redmond, WA, USA). Cytotoxicity analysis was carried out by eight replicates for each plate and three independent experiments. The cell cytotoxicity (%) was calculated by using GraphPad Software 5.0 for Windows (San Diego, CA, USA). In order to check corelation quantitative chemical composition among the different extracts, Pearson’s correlation coefficients were calculated. The probability values below 5% were regarded as significant. For the multivariate projection of samples on the two-dimensional plane, principal component analysis (PCA) was carried out through XLSTAT Statistical Software using Microsoft Excel 2016.

## 5. Conclusions

Identification and quantification of the chemical composition in the essential oils and oleoresins or extracts are important to ensure the quality and safety of raw ingredients for herbal preparation. The documented results imply that acetone extract had the highest extractable solids, followed by ethyl acetate and water, indicating that the yield of extracts could be significantly affected by the solvents. It also indicates that maceration with acetone, ethyl acetate and water were successful techniques for extracting various types of compounds from *A. africana*. Therefore, the chemical compositions and cytotoxic activity are worthwhile to evaluate the toxicity and potential new source of antitumour compounds from the micropropagated *A. africana*. The current cytotoxic action of *A. africana* was much less than the standard drug. The studied cytotoxic effect might be responsible for the predominant components as well as synergistic action of minor components of essential oil and oleoresins. Further studies are required to isolate the dominant components from the micropropagated plants of *A. africana* to determine the exact reason for the cytotoxic activity. Overall, the present investigation would promise future application to the food and herbal industries due to the low toxic effect of the essential oils and oleoresins.

## Figures and Tables

**Table 1:** Chemical composition (%) of the essential oil and oleoresins of *A. africana*.

		Composition (%)		
Compounds	RI	Essential Oil	Acetone Ext.	EtOAc Ext.
***Aliphatic hydrocarbons***				
(1) 2,4,4-Trimethyl-1-hexene	799	0.50		
(2) 2-Hexene, 2,5,5-trimethyl	816	2.12		
(3) 2-Hexene, 3,4,4-trimethyl	816	0.40		
(4) 2,3-Dimethyl-2-heptene	878	0.46		
(5) Cyclopentane, 1,2,3,4,5-pentamethyl	905	0.82		
(6) Nonane, 4,5-dimethyl-	986	0.30		
(7) Octane, 5-ethyl-2-methyl	986	0.63		
(8) n-Decane	1015			0.70
(9) 1-Undecene, 4-methyl	1140			0.63
(10) Dodecane	1214		0.55	0.60
(11) Cyclohexane, (1,2,2-trimethylbutyl)	1228	1.75		
(12) Dodecane, 2,6,11-trimethyl-	1320	1.53		
(13) Tetradecane	1413		6.60	3.83
(14) Pentadecane	1512		0.42	
(15) Hexadecane, 4-methyl	1647		0.35	
(16) Heptadecane	1711	1.17	16.48	9.40
(17) Heptadecane, 7-methyl	1746		0.21	
(18) Octadecane, 4-methyl	1846		0.40	
(19) Nonadecane, 2,3-dimethyl	1980		0.22	
(20) Eicosane	2009	3.89	26.34	6.40
(21) Heneicosane	2109			9.45
(22) 2-methyltetracosane	2442	1.00		
***Aliphatic alcohols***				
(23) 2-Pentanone,4-hydroxy-4-methyl	845		11.13	4.79
(24) cis-4-Hexen-1-ol	868	0.30		
(25) 4,4,6-Trimethyl-cyclohex-2-en-1-ol	1085	0.35		
(26) Spiro[2.4]heptane-5-methanol, 5-hydroxy	1208	0.49		
(27) Pentadecanol	1755		1.12	
(28) 2-Pentadecyn-1-ol	1772	3.77		
(29) Nonadecanol	2153		1.57	0.82
(30) Lignoceric alcohol	2650		1.15	
***Aliphatic aldehyde***				
(31) n-Hexanal	806	0.88		
(32) trans-2-Decenal	1212	0.49		
(33) Mesityl oxide	739		2.34	
***Aliphatic acids and esters***				
(34) n-Butyl acetate	785	-	21.13	14.34
(35) 4-Heptenoic acid, 3,3-dimethyl-6-oxo-methyl ester	1242	0.49		
(36) Myristic acid	1769	0.46		
(37) Pentadecanoic acid	1869	1.31		
(38) Palmitoleic acid	1976	1.42		
(39) Succinic acid, 3,7-dimethyloct-6-en-1-yl pentyl ester	2165	0.68		
(40) Linoleic acid	2183	18.42	2.17	
(41) Linolenic acid	2191	10.98		
(42) *l*-Ascorbyl 2,6-Dipalmitate	4765	11.50	0.46	1.28
***Aromatic hydrocarbons***				
(43) Toluene	794			2.54
(44) Ethylbenzene	893	0.51	0.47	1.34
(45) *p*-Xylene	907	1.53	2.28	5.24
(46) o-Xylene	907		0.70	
(47) 1-Triazene, 1-methyl-3-(4-methylphenyl)	907			0.65
(48) Mesitylene	1020	0.34	0.20	2.98
(49) o-Ethyltoluene	1006			0.78
(50) m-Propyltoluene	1106			0.62
(51) p-Diethylbenzene	1106			1.07
(52) Benzene, 1-ethyl-2,4-dimethyl	1119			0.93
(53) Benzene, 1-ethyl-3,5-dimethyl-	1119			1.15
(54) Durene	1133			1.84
***Aromatic alcohols***				
(55) *p*-Cresol	1014	9.99		
(56) Erythro-1-Phenylpropane-1,2-diol	1317			2.54
***Aromatic aldehydes***				
(57) Styrene	883	4.64		22.20
(58) Benzaldehyde	982	0.39		
***Aromatic ketones***				
(59) Hyacinthin	1081	1.26		
(60) Benzyl methyl ketone	1128	1.64		
***Aromatic acids and esters***				
(61) 2-Ethylbutyric acid, 3-methylbenzyl ester	1606	0.51		
***Aromatic acids and esters***				
(62) Phthalic acid, diisobutyl ester	1908		1.03	
(63) Cyclohexanecarboxylic acid, 4-nitrophenyl ester	2016	0.40		
(64) Phthalic acid, dibutyl ester	2037	0.79	0.44	
(65) Cyclopropanecarboxylic acid, 1-(phenylmethyl)-, 2,6-bis(1,1-dimethylethyl)-4-methylphenyl ester	2775	0.37		
**Monoterpenoides**				
(66) Eucalyptol	1059	1.38		
(67) *β*-Linalool	1082	2.26		
(68) 1,7,7-Trimethyl-2-vinylbicyclo[2.2.1]hept-2-ene	1111	0.68		
(69) p-Menth-1-en-4-ol	1137	0.31		
(70) *α*-Terpineol	1143	1.11		
(71) Dihydroedulan I	1342	0.31		
(72) Dihydroactinidiolide	1426	0.72		
(73) *Trans*-5-Isopropyl-6,7-epoxy-8-hydroxy-8-methyl	1465	0.34		
***Oxygenated norisoprenoids***				
(74) Theaspirane	1370	0.38		
***Sesquiterpenoides***				
(75) Cadalene	1706	0.66		
(76) 3-Isopropyl-6,7-dimethyltricyclo [4.4.0.0(2,8)] decane-9,10-diol	1710	0.52		
(77) Hexahydrofarnesyl acetone	1754	0.30	0.30	
***Azulene molecules***				
(78) Ethanone, 1-(1,3a,4,5,6,7-hexahydro-4-hydroxy-3,8-dimethyl-5-azulenyl)	1758	0.36		
***Diterpenoids***				
(79) Phytol	2045		0.78	
(80) Phytol, acetate	2168		1.16	
(81) Cyclotetrasiloxane, octamethyl	827	0.35		0.91
(82) Cyclopentasiloxane, decamethyl	1034	0.94		2.97
(83) 2,5-Pyrrolidinedione, 3-ethyl-1,3-dimethyl	1326	0.44		
(84) 1,8 (2H,5H)-Naphthalenedione, hexahydro-8a-methyl-, *cis*	1517	0.46		
***Total*** (%)		100	100	100
***Total (number of compounds)***		55	26	26

Note: The retention indices (RI) of compounds determined on Zebron ZB5MS-plus column using GC-FID.

**Table 2:** Extraction yields and cytotoxic activity of essential oil and oleoresins of *A. africana*.

Extract	Extraction Yield (mg/100g)	Cytotoxicity Using Vero Cell Line ^a^ (LC_50_ μg/mL)
Essential oil	10	52.53 ± 0.69
Ethyl acetate extract	5.3	60.05 ± 1.46
Acetone extract	3770	25.64 ± 0.78
Doxorubicin hydrochloride		3.46 ± 0.18 or (5.97 μM/mL)

^a^ Normal adult African green monkey kidney cells. All data represent the mean ± standard deviation. Number of samples: three (n = 3) with eight replicates, the significant difference was calculated at *p* ≤ 0.05.

## Data Availability

The data presented in this study are available on request from the corresponding author.

## Supplementary Materials

The following are available online, Figure S1: GC-MS Chromatogram of essential oil (A), acetone extract (B) and ethyl acetate extract (C).

## Sample Availability

Samples of the compounds are available from the authors.
