# Supplementary material for: Chemical Composition and Cytotoxic Activity of the Essential Oil and Oleoresins of In Vitro Micropropagated Ansellia africana Lindl: A Vulnerable Medicinal Orchid of Africa
Source: Molecules. 2021 Jul 28;26(15):4556. doi: 10.3390/molecules26154556 (PMC8347246; doi:10.3390/molecules26154556)
Supplement: Supplementary file 1 [file molecules-26-04556-s001.zip › molecules-1279385-supplementary.pdf]

molecules

MDPI

Article

Chemical Composition and Cytotoxic Activity of the Essential Oil and Oleoresins of In Vitro Micropropagated *Ansellia africana* Lindl: A Vulnerable Medicinal Orchid of Africa

Md. Moshfekus Saleh-E-In †, Paromik Bhattacharyya and Johannes Van Staden *

Research Centre for Plant Growth and Development, School of Life Sciences, University of KwaZulu-Natal Pietermaritzburg, Private Bag X01, Scottsville 3209, South Africa; saleheinm@kangwon.ac.kr (M.M.S.); paromik600@gmail.com (P.B.)

* Correspondence: rcpgd@ukzn.ac.za; Tel: +27 33 2605130

† Current address: Department of Forest Resources, Kangwon National University, Chuncheon 200-701, South Korea.

Citation: Saleh-E-In, M.M.; Bhattacharyya, P.; Van Staden, J. Chemical composition and cytotoxic activity of the essential oil and oleoresins of in vitro micropropagated *Ansellia africana* Lindl: A vulnerable medicinal orchid of Africa. *Molecules* **2021**, *26*, 4556. https://doi.org/10.3390/molecules26154556

Academic Editor: Ricardo Calhelha

Received: 14 June 2021
Accepted: 20 July 2021
Published: 28 July 2021

Publisher's Note: MDPI stays neutral with regard to jurisdictional claims in published maps and institutional affiliations.

Copyright: © 2021 by the authors. Licensee MDPI, Basel, Switzerland. This article is an open access article distributed under the terms and conditions of the Creative Commons Attribution (CC BY) license (http://creativecommons.org/licenses/by/4.0/).

List of figure

(A) Chromatogram of Essential Oil

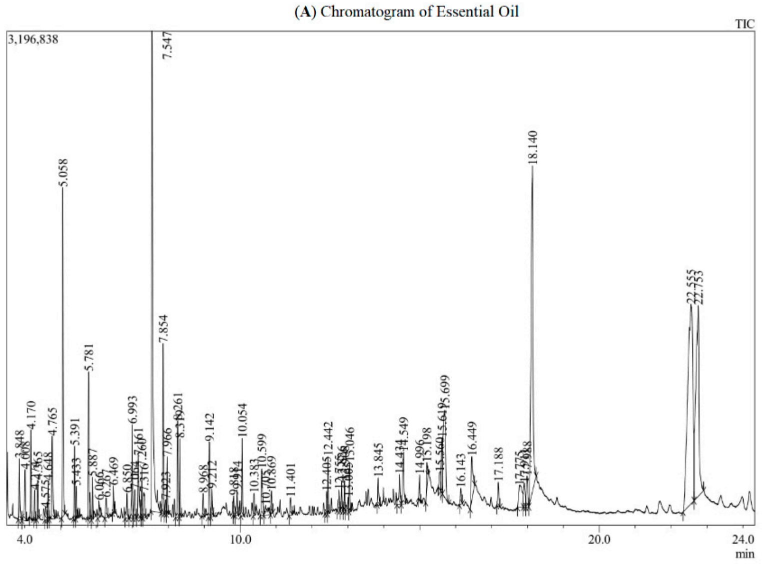

Peak Report TIC

Peak#	R.Time	Area	Area%	Height	Height%	A/H	Name
1	3.848	620273	0.88	394289	1.50	1.57	Hexanal
2	4.008	551270	0.78	330085	1.26	1.67	2-Hexene, 2,5,5-trimethyl-
3	4.170	702009	1.00	574313	2.19	1.22	2-Hexene, 2,5,5-trimethyl-
4	4.274	235902	0.34	189232	0.72	1.25	2-Hexene, 2,5,5-trimethyl-
5	4.365	320509	0.46	252754	0.96	1.27	2,3-Dimethyl-2-heptene
6	4.575	214367	0.30	73903	0.28	2.90	4-Hexen-1-ol, (Z)-
7	4.648	357382	0.51	250595	0.95	1.43	Ethylbenzene
8	4.765	1079062	1.53	536987	2.05	2.01	p-Xylene
9	5.058	3261676	4.64	2130118	8.12	1.53	Styrene
10	5.391	579099	0.82	468252	1.78	1.24	Cyclopentane, 1,2,3,4,5-pentamethyl-
11	5.433	278752	0.40	209602	0.80	1.33	2-Hexene, 3,4,4-trimethyl-
12	5.781	1233884	1.75	949992	3.62	1.30	Cyclohexane, (1,2,2-trimethylbutyl)-
13	5.887	351374	0.50	274085	1.04	1.28	2,4,4-Trimethyl-1-hexene
14	6.066	271640	0.39	108970	0.42	2.49	Benzaldehyde
15	6.267	247843	0.35	128215	0.49	1.93	Cyclotetrasiloxane, octamethyl-
16	6.469	239550	0.34	176962	0.67	1.35	Mesitylene
17	6.850	356562	0.51	160610	0.61	2.22	2-Ethylbutyric acid, 3-methylbenzyl ester
18	6.993	971962	1.38	605450	2.31	1.61	Eucalyptol
19	7.064	345769	0.49	181068	0.69	1.91	4-Heptenoic acid, 3,3-dimethyl-6-oxo-, methyl
20	7.161	886991	1.26	399185	1.52	2.22	Benzeneacetaldehyde
21	7.260	443064	0.63	326688	1.24	1.36	Octane, 5-ethyl-2-methyl-
22	7.316	481029	0.68	162851	0.62	2.95	1,7,7-Trimethyl-2-vinylbicyclo[2.2.1]hept-2-er
23	7.547	7028219	9.99	3105987	11.84	2.26	p-Cresol
24	7.854	1593063	2.26	1097659	4.18	1.45	1,6-Octadien-3-ol, 3,7-dimethyl-
25	7.923	208346	0.30	88174	0.34	2.36	Nonane, 4,5-dimethyl-
26	7.966	479766	0.68	369208	1.41	1.30	Succinic acid, 3,7-dimethyloct-6-en-1-yl penty
27	8.261	1152925	1.64	623868	2.38	1.85	Benzyl methyl ketone
28	8.319	659013	0.94	506358	1.93	1.30	Cyclopentasiloxane, decamethyl-
29	8.968	217609	0.31	146992	0.56	1.48	3-Cyclohexen-1-ol, 4-methyl-1-(1-methylethyl
30	9.142	780852	1.11	485778	1.85	1.61	L-.alpha.-Terpineol
31	9.212	309069	0.44	179527	0.68	1.72	2,5-Pyrrolidinedione, 3-ethyl-1,3-dimethyl-
32	9.818	236912	0.34	125541	0.48	1.89	trans-5-Isopropyl-6,7-epoxy-8-hydroxy-8-metl
33	9.914	341635	0.49	168531	0.64	2.03	2-Decenal, (E)-
34	10.054	718499	1.02	502279	1.91	1.43	Dodecane, 2,6,11-trimethyl-
35	10.383	215347	0.31	142983	0.54	1.51	2H-1-Benzopyran, 3,4,4a,5,6,8a-hexahydro-2,
36	10.599	703605	1.00	301085	1.15	2.34	2-methyltetracosane
37	10.705	356961	0.51	64768	0.25	5.51	Dodecane, 2,6,11-trimethyl-
38	10.869	244909	0.35	149389	0.57	1.64	4,4,6-Trimethyl-cyclohex-2-en-1-ol

Peak#	R.Time	Area	Area%	Height	Height%	A/H	Name
39	11.401	251596	0.36	114873	0.44	2.19	Ethanone, 1-(1,3a,4,5,6,7-hexahydro-4-hydrox
40	12.405	263731	0.37	142539	0.54	1.85	Cyclopropanecarboxylic acid, 1-(phenylmethy
41	12.442	527716	0.75	367948	1.40	1.43	Eicosane
42	12.755	283426	0.40	131500	0.50	2.16	Cyclohexanecarboxylic acid, 4-nitrophenyl est
43	12.826	323420	0.46	191941	0.73	1.68	1,8(2H,5H)-Naphthalenedione, hexahydro-8a-
44	12.908	286292	0.41	197017	0.75	1.45	Eicosane
45	13.005	268235	0.38	91075	0.35	2.95	1-Oxaspiro[4.5]dec-6-ene, 2,6,10,10-tetrametl
46	13.046	507735	0.72	318105	1.21	1.60	2(4H)-Benzofuranone, 5,6,7,7a-tetrahydro-4,4
47	13.845	366345	0.52	176522	0.67	2.08	3-Isopropyl-6,7-dimethyltricyclo[4.4.0.0(2,8)]
48	14.434	463947	0.66	193531	0.74	2.40	Naphthalene, 1,6-dimethyl-4-(1-methylethyl)-
49	14.549	841818	1.20	335849	1.28	2.51	Eicosane
50	14.996	262263	0.37	167494	0.64	1.57	Eicosane
51	15.198	321007	0.46	169100	0.64	1.90	Tetradecanoic acid
52	15.560	344290	0.49	110495	0.42	3.12	Spiro[2.4]heptane-5-methanol, 5-hydroxy-
53	15.619	820210	1.17	355411	1.35	2.31	Heptadecane
54	15.699	2652147	3.77	565515	2.15	4.69	2-Pentadecyn-1-ol
55	16.143	214199	0.30	104537	0.40	2.05	2-Pentadecanone, 6,10,14-trimethyl-
56	16.449	924926	1.31	285815	1.09	3.24	Pentadecanoic acid
57	17.188	368633	0.52	155608	0.59	2.37	Eicosane
58	17.775	995551	1.42	137870	0.53	7.22	Palmitoleic acid
59	17.911	451474	0.64	154904	0.59	2.91	Eicosane
60	17.988	555015	0.79	199885	0.76	2.78	Dibutyl phthalate
61	18.140	8093213	11.50	2105824	8.02	3.84	l-(+)-Ascorbic acid 2,6-dihexadecanoate
62	22.555	12961627	18.42	1290816	4.92	10.04	9,12-Octadecadienoic acid (Z,Z)-
63	22.753	7727853	10.98	1235928	4.71	6.25	9,12,15-Octadecatrienoic acid, (Z,Z,Z)-
		70353368	100.00	26242435	100.00		

(B) Chromatogram of acetone extract

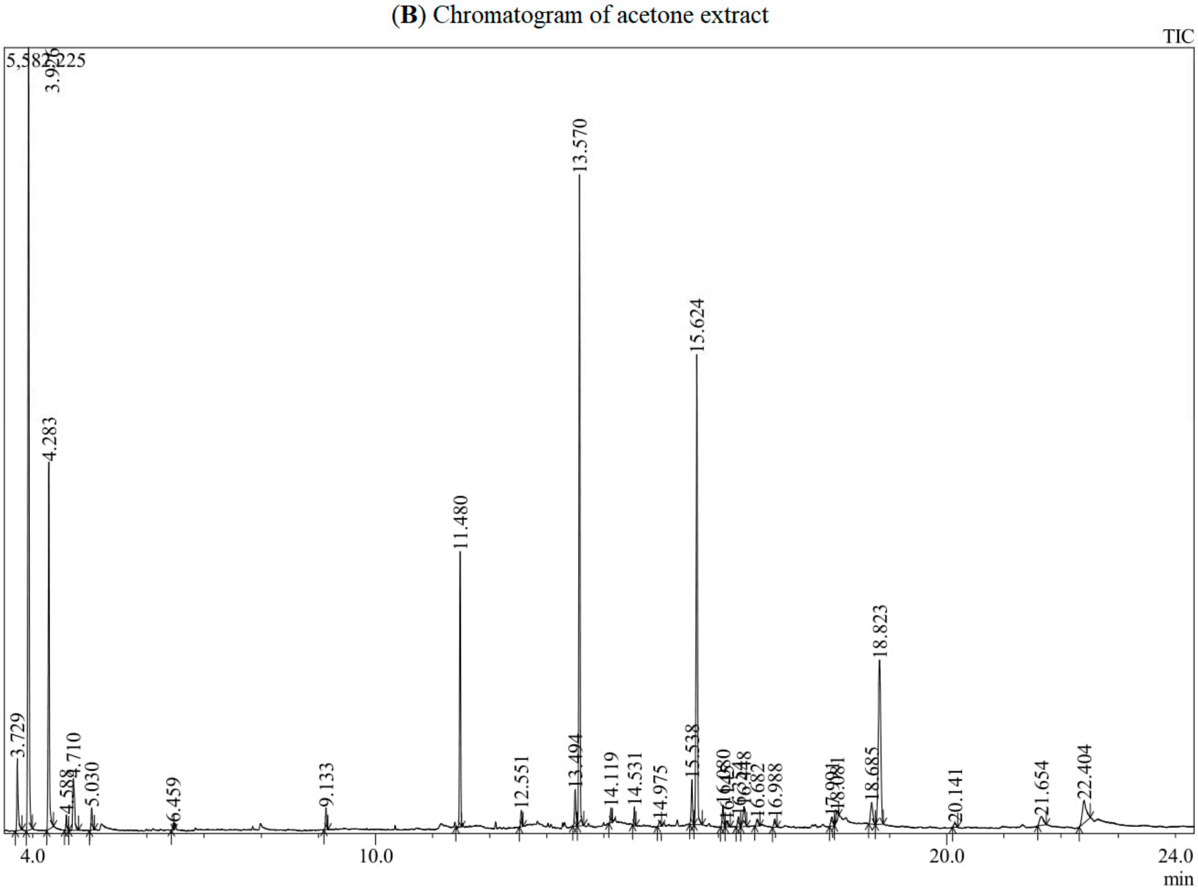

Peak Report TIC

Peak#	R.Time	Area	Area%	Height	Height%	A/H	Name
1	3.729	841844	2.34	511162	2.26	1.65	3-Penten-2-one, 4-methyl-
2	3.926	7594352	21.13	5558745	24.56	1.37	Acetic acid, butyl ester
3	4.283	4000004	11.13	2605597	11.51	1.54	2-Pentanone, 4-hydroxy-4-methyl-
4	4.588	170046	0.47	109491	0.48	1.55	Ethylbenzene
5	4.710	818497	2.28	368263	1.63	2.22	p-Xylene
6	5.030	250961	0.70	158673	0.70	1.58	o-Xylene
7	6.459	71317	0.20	50345	0.22	1.42	Mesitylene
8	9.133	196481	0.55	154919	0.68	1.27	Dodecane
9	11.480	2373814	6.60	1955744	8.64	1.21	Tetradecane
10	12.551	151145	0.42	117912	0.52	1.28	Pentadecane
11	13.494	402213	1.12	261088	1.15	1.54	n-Pentadecanol
12	13.570	5751774	16.00	4627734	20.45	1.24	Heptadecane
13	14.119	126357	0.35	105636	0.47	1.20	Hexadecane, 4-methyl-
14	14.531	170941	0.48	130721	0.58	1.31	Heptadecane
15	14.975	76155	0.21	40779	0.18	1.87	Heptadecane, 7-methyl-
16	15.538	562717	1.57	317993	1.41	1.77	n-Nonadecanol-1
17	15.624	5827639	16.21	3340375	14.76	1.74	Eicosane
18	16.080	302131	0.84	154181	0.68	1.96	Phytol, acetate
19	16.145	108274	0.30	42277	0.19	2.56	2-Pentadecanone, 6,10,14-trimethyl-
20	16.354	143997	0.40	71721	0.32	2.01	Octadecane, 4-methyl-
21	16.448	368663	1.03	140814	0.62	2.62	1,2-Benzenedicarboxylic acid, bis(2-methylprc
22	16.682	113257	0.32	49849	0.22	2.27	Phytol, acetate
23	16.988	128868	0.36	61996	0.27	2.08	Eicosane
24	17.991	158691	0.44	63657	0.28	2.49	Dibutyl phthalate
25	18.081	165175	0.46	55963	0.25	2.95	l-(+)-Ascorbic acid 2,6-dihexadecanoate
26	18.685	414272	1.15	155903	0.69	2.66	n-Tetracosanol-1
27	18.823	3510809	9.77	1165418	5.15	3.01	Eicosane
28	20.141	80075	0.22	29378	0.13	2.73	Nonadecane, 2,3-dimethyl-
29	21.654	282074	0.78	62617	0.28	4.50	Phytol
30	22.404	780570	2.17	160217	0.71	4.87	9,12-Octadecadienoic acid (Z,Z)-

(C) Chromatogram of ethyl acetate extract

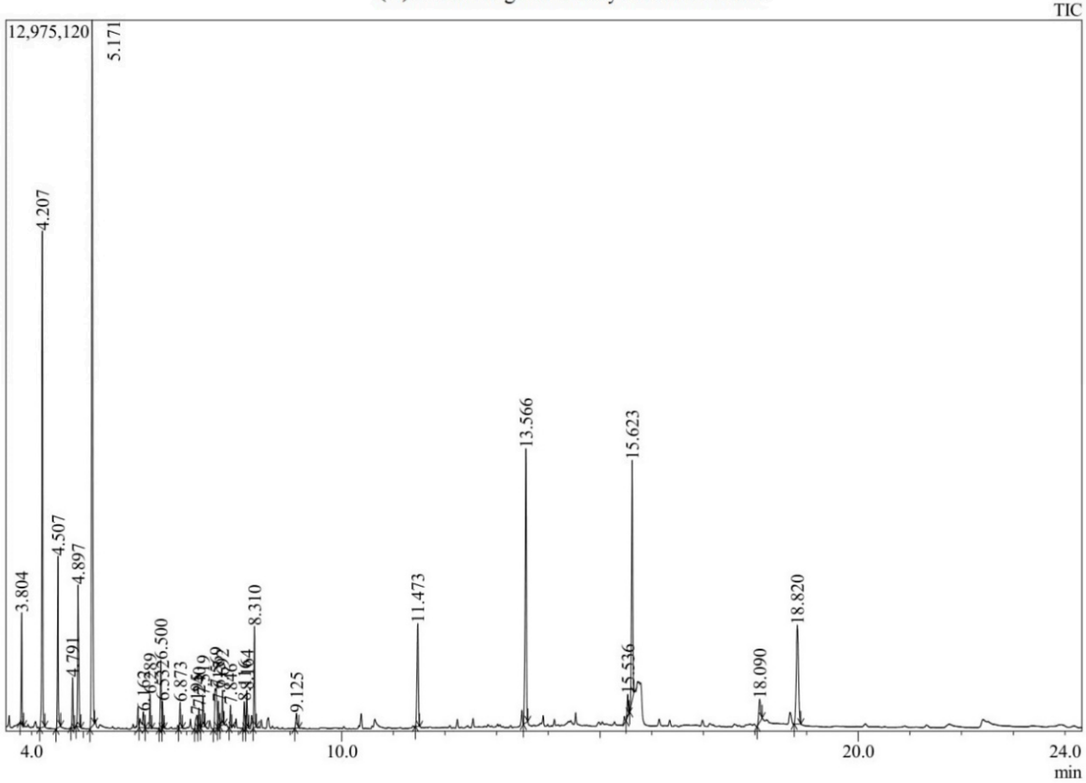

Peak Report TIC

Peak#	R.Time	Area	Area%	Height	Height%	A/H	Name
1	3.804	2108935	2.54	2027380	3.74	1.04	Toluene
2	4.207	11910981	14.34	9055995	16.71	1.32	Acetic acid, butyl ester
3	4.507	3976511	4.79	3126780	5.77	1.27	2-Pentanone, 4-hydroxy-4-methyl-
4	4.791	1109387	1.34	915732	1.69	1.21	Ethylbenzene
5	4.897	4355171	5.24	2578857	4.76	1.69	p-Xylene
6	5.171	18440978	22.20	12881855	23.76	1.43	Styrene
7	6.162	802559	0.97	289481	0.53	2.77	Mesitylene
8	6.289	754527	0.91	616150	1.14	1.22	Cyclotetrasiloxane, octamethyl-
9	6.500	1670407	2.01	1247082	2.30	1.34	Mesitylene
10	6.532	579136	0.70	489470	0.90	1.18	Decane
11	6.873	643861	0.78	464129	0.86	1.39	Benzene, 1-ethyl-2-methyl-
12	7.195	541152	0.65	238848	0.44	2.27	1-Triazene, 1-methyl-3-(4-methylphenyl)-
13	7.240	512000	0.62	361072	0.67	1.42	Benzene, 1-methyl-3-propyl-
14	7.319	886162	1.07	556302	1.03	1.59	Benzene, 1,4-diethyl-
15	7.569	2138508	2.57	725809	1.34	2.95	dl-Erythro-1-phenyl-1,2-propanediol
16	7.615	770846	0.93	439982	0.81	1.75	Benzene, 1-ethyl-2,4-dimethyl-
17	7.692	958088	1.15	633098	1.17	1.51	Benzene, 1-ethyl-3,5-dimethyl-
18	7.846	522461	0.63	406156	0.75	1.29	1-Undecene, 4-methyl-
19	8.116	626331	0.75	486050	0.90	1.29	Benzene, 1,2,4,5-tetramethyl-
20	8.164	902805	1.09	681618	1.26	1.32	Benzene, 1,2,4,5-tetramethyl-
21	8.310	2464696	2.97	1839911	3.39	1.34	Cyclopentasiloxane, decamethyl-
22	9.125	501944	0.60	271414	0.50	1.85	Dodecane
23	11.473	3180179	3.83	1870827	3.45	1.70	Tetradecane
24	13.566	7810397	9.40	4997475	9.22	1.56	Heptadecane
25	15.536	682552	0.82	408700	0.75	1.67	n-Nonadecanol-1
26	15.623	7850027	9.45	4369683	8.06	1.80	Heneicosane
27	18.090	1062750	1.28	425977	0.79	2.49	l-(+)-Ascorbic acid 2,6-dihexadecanoate
28	18.820	5315373	6.40	1803382	3.33	2.95	Eicosane
		83078724	100.00	54209215	100.00		

Figure S1. GC-MS Chromatogram of (**A**) essential oil, (**B**) acetone extract and (**C**) ethyl acetate extract.
